# Silver nanoparticles (AgNPs) cause degeneration of cytoskeleton and disrupt synaptic machinery of cultured cortical neurons

**DOI:** 10.1186/1756-6606-6-29

**Published:** 2013-06-19

**Authors:** Fenglian Xu, Cortt Piett, Svetlana Farkas, Munir Qazzaz, Naweed I Syed

**Affiliations:** 1 Department of Cell Biology & Anatomy, Hotchkiss Brain Institute, Faculty of Medicine, University of Calgary, Calgary, Alberta, Canada; 2 Department of Physiology & Pharmacology, Hotchkiss Brain Institute, Faculty of Medicine, University of Calgary, Calgary, Alberta, Canada; 3 Department of Biology, Mount Royal University, Calgary, Alberta, Canada; 4 Faculty of Nursing and Allied Health Professions, Birzeit University, Birzeit, West Bank, Palestine

**Keywords:** Silver nanoparticles, Rat cortical culture, Toxicity, Cytoskeleton, Synaptic machinery, Mitochondria

## Abstract

**Background:**

Silver nanoparticles (AgNPs), owing to their effective antimicrobial properties, are being widely used in a broad range of applications. These include, but are not limited to, antibacterial materials, the textile industry, cosmetics, coatings of various household appliances and medical devices. Despite their extensive use, little is known about AgNP safety and toxicity vis-à-vis human and animal health. Recent studies have drawn attention towards potential neurotoxic effects of AgNPs, however, the primary cellular and molecular targets of AgNP action/s remain to be defined.

**Results:**

Here we examine the effects of ultra fine scales (20 nm) of AgNPs at various concentrations (1, 5, 10 and 50 μg/ml) on primary rat cortical cell cultures. We found that AgNPs (at 1-50 μg/ml) not only inhibited neurite outgrowth and reduced cell viability of premature neurons and glial cells, but also induced degeneration of neuronal processes of mature neurons. Our immunocytochemistry and confocal microscopy studies further demonstrated that AgNPs induced the loss of cytoskeleton components such as the β-tubulin and filamentous actin (F-actin). AgNPs also dramatically reduced the number of synaptic clusters of the presynaptic vesicle protein synaptophysin, and the postsynaptic receptor density protein PSD-95. Finally, AgNP exposure also resulted in mitochondria dysfunction in rat cortical cells.

**Conclusions:**

Taken together, our data show that AgNPs induce toxicity in neurons, which involves degradation of cytoskeleton components, perturbations of pre- and postsynaptic proteins, and mitochondrial dysfunction leading to cell death. Our study clearly demonstrates the potential detrimental effects of AgNPs on neuronal development and physiological functions and warns against its prolific usage.

## Background

Nanoparticles are ultra-fine materials (range of 1-100 nm in length or diameter) that have gained enormous popularity in modern technology, medical health care, and commercial products [1-3]. Silver nanoparticles (AgNPs) are one of the most commonly used metal-nanoparticles, which possess potent antibacterial and antifungal characteristics. AgNPs have been used extensively as an antimicrobial agent in cosmetics, textiles and the food industry, as well as a disinfectant for medical devices and for coating home applicance [4]. AgNPs upon entering the human body can be systemically distributed throughout, and may affect organs like the lung, liver, spleen, kidney and the central nervous system (CNS) [5-7]. Although various organs can rid themselves of AgNPs, these particles tend to reside for a considerable time, and exhibit a longer half-life within the brain than in other organs [8]. AgNPs could gain access to the CNS through the upper respiratory tract via the olfactory bulb [9] or through the blood–brain barrier (BBB) [5,8,10] and accumulate in various brain regions [4,11]. AgNPs are also known to cause inflammation and disruption of the BBB [12]. Although the translocation of AgNPs into the brain through the BBB is fairly low under normal condition, its accumulation is augmented under pathological conditions such as meningitis, stroke, or systemic inflammation [8,13]. Therefore, there exist potential health risks within the brain when exposed to, or upon consumption of AgNP-containing substances.

In the past several years, researchers have begun to explore the potential neurotoxicity of AgNPs using animal models and primary neuronal cell cultures. For instance, studies have reported that animals treated with AgNPs exhibited cognitive impairment, motor deficits and cellular alterations in the brain [8]. In AgNP-treated zebrafish embryos, AgNPs have been found to mainly distribute in the brain, heart, and the blood. Accordingly, AgNPs resulted in cardiorespiratory arrhythmicity, slow blood flow, and impaired body movement and development [14,15]. In mixed primary neuronal cell cultures of mouse frontal cortex, AgNPs have been found to induce acute intracellular calcium rise followed by a strong oxidative stress response and cytotoxicity in both neurons and glial cells [16]. Glial cells were found in this study to be more vulnerable to AgNP toxicity than neurons. Other studies have revealed that AgNPs could alter excitatory glutamatergic synaptic transmission and receptor functions [16]. It could also change cellular excitability by affecting voltage-gated sodium [17] and potassium channels [18] in primary CA1 neurons from mice.

Despite the potential effects of AgNP neurotoxicity cited above, it is still not known whether these nanoparticles could differentially affect brain tissues in the early developmental stage versus later growth phases. It is also unclear whether AgNPs affect fundamental structural and functional components such as the cytoskeleton, mitochondria and synaptic machinery. In the present study, we first examined the effect of AgNPs on neurite outgrowth and cell viability during both early (< 6 days in culture) and more mature (> 10 days) developing stages. We found that AgNPs (20 nm) reduced cell viability in both the early and later stage of cultures in a concentration-dependent manner. Specifically, AgNPs not only inhibited the sprouting of neuronal branches and elongation of neurites, but also caused fragmentation and degeneration of mature neurons. Our data further demonstrated that AgNP neurotoxicity involves the perturbation of structural and/or functional integrity of cytoskeletal components, mitochondria and synaptic proteins.

## Results

### AgNPs inhibit neuronal extension, neuritic overlap, and compromise cell viability of cultured rat cortical cells

**Figure 1:**
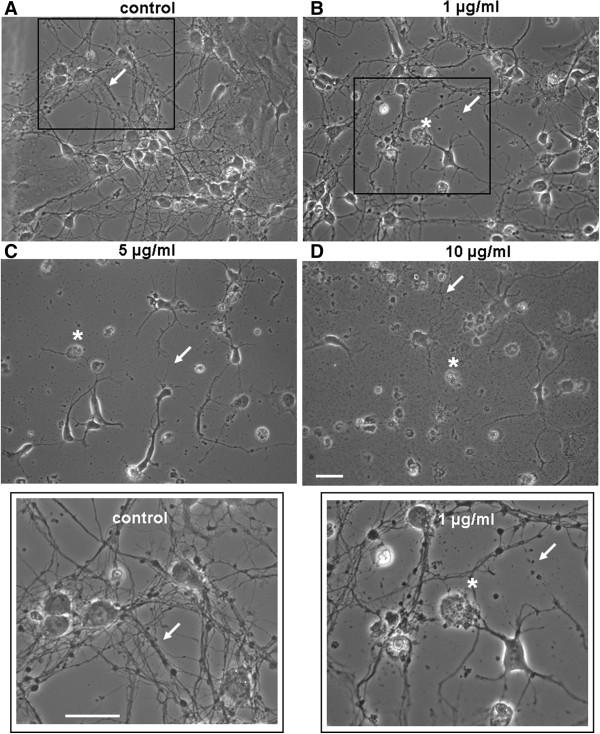
**Exposure to AgNPs compromises neuronal viability and perturbs neurite outgrowth.** Phase contrast images of rat cortical neurons cultured either in the absence (**A**, control) or presence of various concentrations of AgNPs at 1 (**B**), 5 (**C**), and 10 (**D**) μg/ml for 3 days. (**A**) Shows the control group grown in the absence of AgNPs is characterized by healthy cell bodies and well-established neuronal processes (indicated by an arrow, also see insert). (**B**) Shows that AgNPs at the concentration of 1 μg/mL caused cell damage (indicated by an asterisk) and triggered fragmentation of developing neuronal networks (indicated by an arrow) (also see insert). (**C**) Indicates that cells cultured in AgNPs at 5 μg/mL have an increased number of deteriorating cells (indicated by an asterisk) and a markedly reduced degree of neurite outgrowth (indicated by an arrow). (**D**) In the presence of AgNPs at 10 μg/mL, the majority of cells underwent cell death and abnormal/limited neuronal sprouting and outgrowth. Arrows indicate neuronal processes and asterisks indicate neuronal cell bodies. Scale bar, 25 μm.

**Figure 2:**
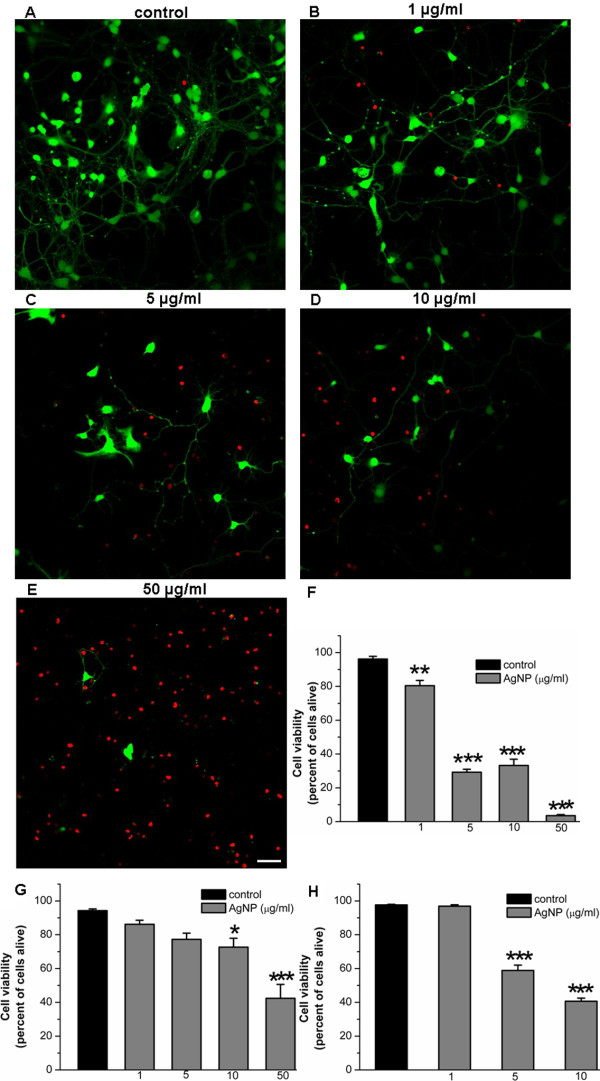
**The effect of AgNPs on cell viability of a developing and mature cortical culture.** To assess the effect of AgNPs on cell viability at the early stages of neuronal development, freshly isolated rat cortical cells were cultured either in the absence (control) or presence of AgNPs at doses ranging from 1 μg/ml to 50 μg/ml. The cell viability/cytotoxicology assay was performed on day 3. The number of live and dead cells in randomly chosen areas of 1 mm^2^ were counted using the imageJ program. The representative images are shown in **A**-**E** and the statistical data of cell viability is presented in the bar graphs of F. Live cells are represented by the green fluorescence of calcein labeling of cell cytosol and neurites, while dead cells are represented by the red fluorescent ethidium homodimer-1 indicating membrane damage. **A**-**F** reveals that when cells were cultured in the presence of AgNPs, their viability and axonal outgrowth was decreased by AgNPs in a concentration-dependent manner. To study how AgNPs affected cell viability at more developed stages, neurons and glia were first cultured in control medium for 4 days (**G**) or 10 days (**H**) respectively, and both groups of cells were subsequently exposed to various concentrations of AgNPs for another 2 days. The Live-dead cell assay was performed 2 days later. G-H show that AgNPs at concentrations greater than the 5 μg/ml significantly reduced cell viability in both culture conditions. Statistical significance was determined using ANOVA one-way analysis of variance. Post hoc analysis was conducted using Tukey’s test. * *P* < 0.05. ** *P* < 0.01. *** *P* < 0.001. Error bars indicate SEM for all figures. Scale bar, 25 μm.

To examine the effect of AgNPs on brain cell development, we first investigated whether AgNPs affect neurite initiation, extension and neuritic overlap. To this end, cells were cultured either in the absence or presence of AgNPs at concentrations of 1, 5, 10, and 50 μg/ml for 3 days. Representative phase contrast images (Figure 1A-1D) were taken on day 3 to evaluate the effect of AgNPs on the development of neurite processes, branches, and overlaps. A Live/Dead cell assay (see Methods) was subsequently performed the same day to evaluate the effect of AgNPs on cell survival. Figure 1A shows that in the absence of AgNPs (as control), cells exhibit healthy cell bodies, extensive branches and overlap (indicated by arrows, also see insert). Despite the fact that cells cultured in the presence of AgNPs at a concentration of 1 μg/ml still form overlaps, the number and degree of cell overlaps were apparently not as extensive when compared to control (Figure 1B). Note that a subpopulation of cell bodies underwent cell death (indicated by asterisks) and some of the neuronal processes appeared fragmented (indicated by an arrow) (Figure 1B, also see insert). Increasing concentrations of AgNPs to 5 and 10 μg/ml augmented the detrimental effect of AgNPs on cell death (indicated by asterisks) and severely diminished the extension of neurites and the degree of neuritic overlaps (indicated by arrows) (Figure 1C and 1D). To further quantify the effect of AgNPs on cell viability, we subsequently performed a Live/Dead cell assay on cells cultured either in the absence or presence of AgNPs for 3 days. To do this, cells were incubated using the Viability ⁄ Cytotoxicity Kit (Invitrogen) for 15 mins at room temperature. Fluorescent images of live and dead cells were taken using a confocal microscope (LSM-510) and images were acquired through a 20× objective under same confocal parameter settings (Figure 2A-2E). Cell viability was indicated by an active enzymatic conversion of the non-fluorescent calcein-AM to the green fluorescent calcein in healthy cells (live cells), and concurrent uptake of the red fluorescent ethidium homodimer-1 through damaged plasma membrane (dead cells). The numbers of live (green) and dead (red) cells under the above culture conditions were counted using imageJ software (Figure 2F). Specifically, cells were counted based on a randomized selection of four to five areas of 1 mm^2^ under each culture condition. The mean value of cell viability reflected by the percentage of live cells was calculated and compared. Figure 2F shows the statistic data demonstrating the effect of AgNPs on cell viability under the control culture condition and in the presence of different concentrations of AgNPs. Our data show that AgNPs at all the concentrations examined significantly compromised cell viability as compared to control (P < 0.05). Specifically, the percent of live cells under control conditions was 96.2 ± 1.6% (n = 4). It was significantly reduced to 80.5 ± 3.1% (n = 4) by 1 μg/ml of AgNPs, to 29.2 ± 1.8% by 5 μg/ml of AgNPs, to 33.2 ± 3.7% by 10 μg/ml of AgNPs, to 3.5 ± 0.7% by 50 μg/ml of AgNPs (Figure 2F).

**Figure 3:**
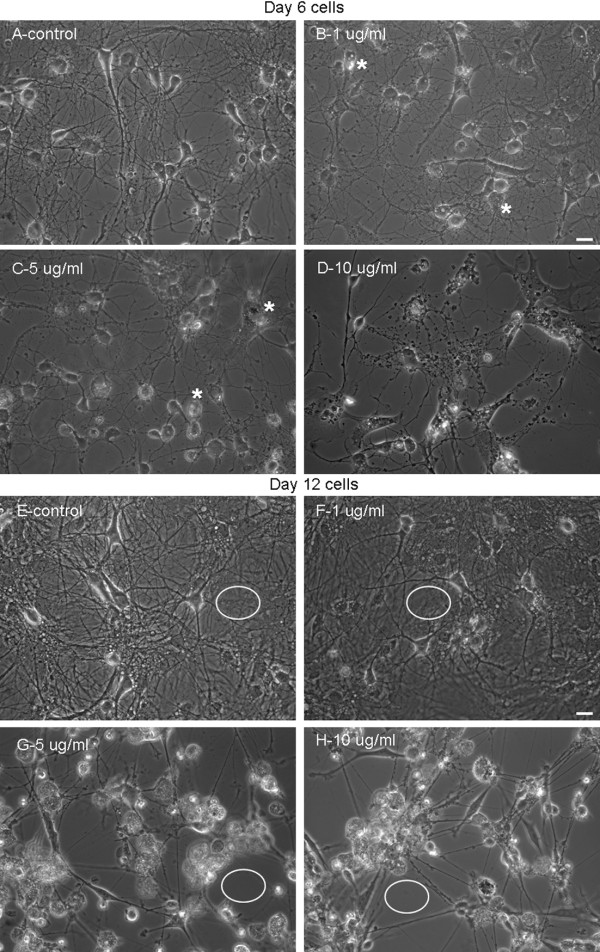
**Exposure to AgNPs compromises neuronal viability and degenerates neuronal processes in well-established cells and network.** To assess whether AgNPs could cause neurotoxicity of cells that had established neurite processes during an early stage of development, rat cortical cultures were first maintained in control culture medium for 4 days and allowed to develop processes and overlaps. Cultures were subsequently exposed to culture medium containing AgNPs at 0 (control-**A**), 1 (**B**), 5 (**C**), and 10 (**D**) μg/ml for 2 days. Phase contrast pictures were taken on day 6. To assess the consequence of AgNP exposure at a later stage of development, rat cortical cultures were first maintained in control culture medium for 10 days and allowed to develop a mature, well-endowed network; cells were subsequently exposed to culture medium containing AgNPs at 0 (control-**E**), 1 (**F**), 5 (**G**), and 10 (**H**) μg/ml for another 2 days. Phase contrast pictures were taken on day 12. Under both culture conditions, AgNPs at high concentrations (> 5 μg/ml) compromised cellular membrane integrity and induced neurite degeneration. It is however interesting to note that AgNPs induced fragmentation of neurites at the early stage of cell culture (**C**-**D**), while triggered an aggregation of cells bodies and thinning of neurite processes in the later stage of cell culture (**G**-**H**). In addition, glial cell layers (indicated by white circles) were present under the control (**E**) and 1 μg/ml (**F**) of AgNP- treated cultures, but were absent in cultures that were exposed to AgNPs at the concentrations of 5 (**G**) and 10 (**H**) μg/ml. Scale bar, 25 μm.

We next asked the question whether AgNPs affect neuronal outgrowth that had been fully established in the early stage of development (< 6 days). To answer this, rat cortical neurons were first maintained in culture for 4 days to allow extensive neuritic development. On day 4, cells were exposed to culture medium only (as control) or culture medium containing AgNPs at the above concentrations. Cells were maintained in culture for another 2 days. Phase contrast images (Figure 3A-3D) were taken on day 6 followed by the Live/Dead cell assay (Figure 2G). Specifically, control culture again yielded healthy populations of cell bodies with extensive interconnective processes (Figure 3A). Although cells exposed to AgNPs at lower concentrations of 1 (Figure 3B) and 5 (Figure 3C) μg/ml still exhibited extensive processes and overlap, the majority of their cell bodies started to show vacuoles, a typical sign for apoptosis (indicated by asterisks). AgNPs, at the concentration of 10 μg/ml, not only severely compromised the morphological integrity of cell bodies, but also drastically caused degeneration of neurites (Figure 3D). Accordingly, AgNPs at 1 and 5 μg/ml reduced cell viability from control level of 94.3 ± 1.0% to 86.2 ± 2.4% (n = 5, P > 0.05) and to 77.3 ± 3.6% (n = 5, P > 0.05), respectively (Figure 2G). However, AgNPs significantly reduced cell viability from the control level of 94.3 ± 1.0% to 72.7 ± 5.3% (n = 5, P < 0.05) by AgNPs at 10 μg/ml and to 42.4 ± 8.2% (n = 5, P < 0.05) by AgNPs at 50 μg/ml (n = 5, P < 0.001) (Figure 2G).

**Figure 4:**
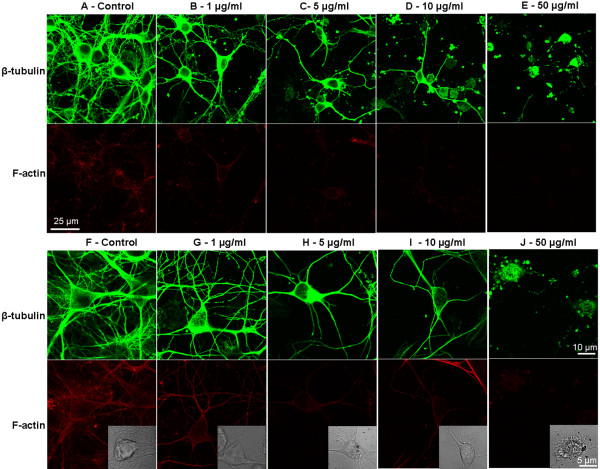
**AgNPs induce reduction or alteration of cytoskeletal components, β-tubulin and F-actin, in rat cortical primary cultures.** We first assessed the effect of AgNPs on neuronal cytoskeleton initiation and outgrowth in cells cultured either in the absence (control) or presence of different concentrations of AgNPs for 3 days (**A**-**E**, via a 20× objective). We also examined whether AgNPs could affect well-established cytoskeleton network in cells that have been cultured for 4 days and were then exposed to various concentrations of AgNPs (**F**-**J**, via a 63× oil objective). In both sets of experiments, control neurons displayed high fluorescence intensity of β-tubulin (green) and F-actin (red), which labeled healthy cell bodies and dense neuritic overlaps. AgNP-treatment increased cell death in combination with a reduction in β-tubulin branching and overlapping (also see inserted phase contrast images). AgNPs also reduced the degree and intensity of red fluorescent labeling of F-actin in AgNP-treated cultures.

Finally, we sought to determine whether mature neurons and their outgrowing patterns (> 10 days) could be impacted after exposure to AgNPs. To examine this, rat cortical cells were first cultured for 10 days. Neurons were then treated with the carrier solution or carrier solution plus AgNPs at various concentrations for another 2 days. The effects of AgNPs were evaluated again by phase contrast pictures (Figure 3E-3H) in combination with the Live/Dead cell assay (Figure 2H). At this stage in the primary cell culture, neurons were fully developed and the glial cells actively formed solid layers (as indicated by white open circles) in the culture dish that surrounded and supported neurons and their neurites. It was obvious that AgNPs, at 1 μg/ml, reduced the intensity of neuronal branches and overlaps. Surprisingly, AgNPs at 5 and 10 μg/ml, caused aggregation of cell bodies into clusters, disruption of neurite processes and damage of glial layers – indicated by the absence of glial layers in the culture dish (see white open circles). In this case, disturbance of neurite connections was not predominately observed as fragmentation of neurites, as seen in the early stage of cell culture (Figure 4A-4D). Interestingly, the damage to neurons and glia seemed to also result from mechanical insult during cell aggregation. The Live/Dead cell assay showed that AgNPs at both of 5 μg/ml (58.9 ± 3.2%, n = 5, P < 0.001) and 10 μg/ml (40.65 ± 1.8%, n = 5, P < 0.001), but not 1 μg/ml (96.9 ± 0.8%, n = 5, P > 0.05) significantly reduced cell viability as compared to control (97.6 ± 0.4%, n = 5) (Figure 2H).

Our data thus demonstrate that AgNPs not only inhibit neuritic initiation, extension and overlap, but also cause degeneration of well-established neuronal structures leading to cell death.

### AgNPs induce alterations in cytoskeletal components in rat cortical neurons

**Figure 5:**
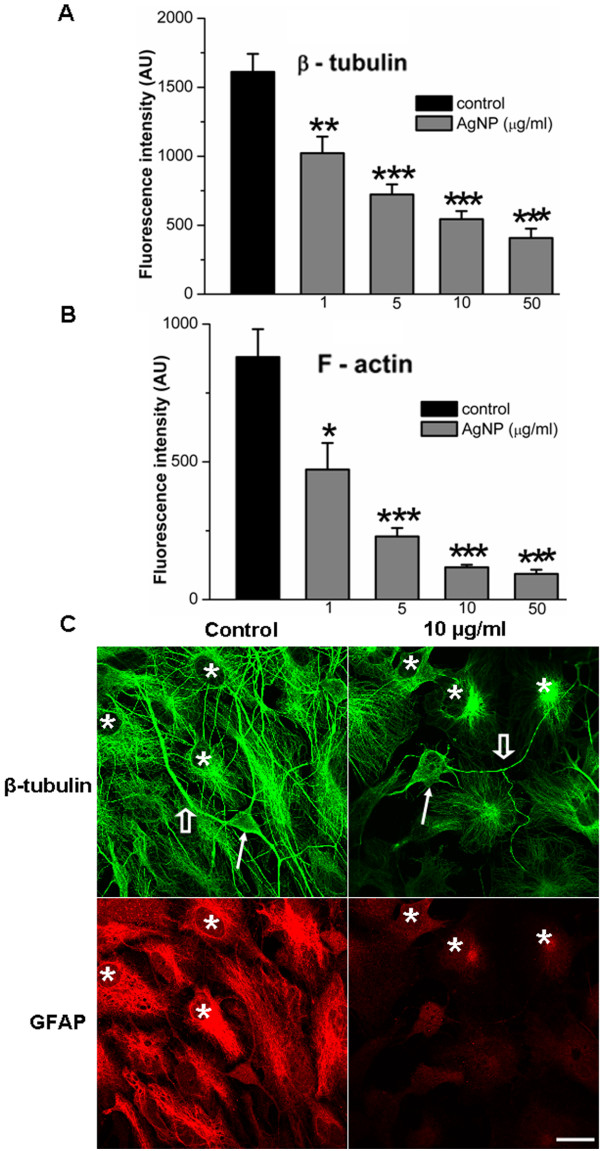
**Statistical data showing the effect of AgNPs on the mean fluorescent intensity of cytoskeletal proteins and representative images showing the impact of AgNPs on glial β-tubulin and GFAP.** To quantify the effect of AgNPs on the level of β-tubulin and F-actin, the fluorescent intensity of randomly selected 7-16 areas of 3844 μm^2^ under control and AgNP-treated conditions (as shown in Figure 4F-4J) were measured using imageJ software. The quantified data show that AgNPs significantly decreased the fluorescent intensity of both β-tubulin (**A**) and F-actin (**B**). To further investigate whether AgNPs affected glial cytoskeleton, cells were co-stained with β-tubulin (green) and GFAP (red), a specific marker for glial astrocytes. **C** shows the representative images of cells in the control and AgNPs at 10 μg/ml. Neurons under the control conditions exhibited healthy neuronal cell bodies (indicated by a solid arrow) and neurites (indicated by an open arrow), while neurons exposed to 10 μg/ml of AgNPs had degenerated cell bodies (indicated by a solid arrow) and neurites (indicated by an open arrow). Note that both of the β-tubulin and GFAP in glial cells (indicated by asterisks) were compromised by AgNPs. * P < 0.05. ** P < 0.01. *** P < 0.001. Error bars indicate SEM. Scale bar, 20 μm.

The remarkable changes in cell morphology and viability induced by AgNPs raised the question as to which primary structures could likely be affected by AgNPs. Because the integrity of cytoskeletal components, mainly the actin filament (F-actin) and the microtubule β-tubulin, are crucial to cell survival, neurite outgrowth, network formation and maintenance, their dynamic assembly/disassembly is very sensitive to subtle chemical changes of the extracellular milieu. The neurotoxicity of AgNPs on brain cell morphological integrity led us to postulate that AgNPs may affect the assembly/disassembly of cytoskeletal components. To test this hypothesis, neurons were first cultured either in the absence or presence of various concentrations of AgNPs for 4 days. Cells were then fixed and stained with β-tubutin antibody and rhodamine-phalloidin, two commonly utilized markers for the study of the integrity of neuronal cytoskeletal structures. The fluorescence labeling of β-tubulin (green) and F-actin (red) were visualized using the confocal LSM 510 microscope and images were collected using the 20× objective under the same parameter settings. Figure 4A shows that cells cultured under control conditions exhibit a high number of healthy neurons with a complex neuronal overlap and extensive fluorescence labeling with both of β-tubulin and F-actin antibodies. AgNP treatment reduced not only the number of healthy cells, but also neuritic branches/overlap that were labeled with F-actin and β-tubulin antibodies in a concentration-dependent manner (Figure 4A-E, top two panels). To further determine whether AgNPs affect cytoskeletal assembly/disassembly status in well-established neurons and their neuritic processes, we first cultured neurons for 4 days and allowed them to establish neurite processes and overlaps. Cells were then maintained in culture medium either lacking (control) or containing different concentrations of AgNPs for another 3 days. Cells were subsequently fixed and immunostained with either β-tubulin/F-actin or β-tubulin/glial fibrillary acidic protein (GFAP) antibodies in order to specifically label glial cells. Figure 4F-4J (bottom two panels) show that cells cultured under the control condition revealed extensive staining of both neuronal β-tubulin (green) as well as the surrounding glial structures. There was also high intensity of F-actin (red) staining of these structures in control cultures (Figure 4F). AgNP-treatment caused apparent deficits in neuronal processes and cell body integrity (Figure 4G-4J). To quantify the effect of AgNPs on the amount of cytoskeletal proteins, the fluorescent intensity of β-tubulin and F-actin in randomly selected areas of 3844 μm^2^ under each condition (as shown in Figure 4F-J) from four repeated experiments was measured using imageJ software. Our statistic data show that AgNPs significantly reduced the mean fluorescent intensity of both the β-tubulin (Figure 5A) and F-actin (Figure 5B) at the concentrations examined. Specifically, AgNPs at 1 μg/ml did not cause noticeable changes in neuronal morphological integrity (Figure 4G), it did however reduce the fluorescent intensity of β-tubulin from the control level of 1611.6 ± 130.9 AU (n = 7) to 1022.1 ± 119.8 AU (n = 11) (P = 0.003), and F-actin from the control level of 880.4 ± 100.8 AU (n = 7) to 471.8 ± 96.7 AU (n = 11) (P = 0.0128). AgNPs at concentrations above 5 μg/ml induced the apparent degeneration of neuronal structures and dissolution of F-actin and β-tubulin proteins (Figure 4H-J). Specifically, the fluorescent intensity of β-tubulin was significantly reduced from the control level of 1611.6 ± 130.9 AU (n = 7) to 723.0 ± 73.1 AU (n = 16, P < 0.001) by 5 μg/ml of AgNPs, to 544.1 ± 58.5 AU (n = 14, P < 0.001) by 10 μg/ml of AgNPs, and to 407.8 ± 67.4 AU (n = 8, P < 0.001) by 50 μg/ml of AgNPs (Figure 5A). The fluorescent intensity of F-actin was significantly reduced from the control level of 880.4 ± 100.8 AU (n = 7) to 229.2 ± 30.7 AU (n = 16, P < 0.001) by 5 μg/ml of AgNPs, to 117.2 ± 9.2 AU (n = 14, P < 0.001) by 10 μg/ml of AgNPs, and to 93.4 ± 14.9 AU (n = 8, P < 0.001) by 50 μg/ml of AgNPs (Figure 5B).

We next examined whether AgNPs affect glial cytoskeletal components. To do this, cells were first cultured for 4 days and were then exposed to AgNPs at different concentrations. On day 7, control and AgNP-treated cultures were immunostained with antibodies of β-tubulin (green) and GFAP (red), a fluorescent marker that specifically labels astrocytes. Our representative fluorescent images in Figure 5C show that under the control conditions, healthy neuronal soma (indicated by a solid arrow) and neurites (indicated by an open arrow) were surrounded by glial cells (indicated by asterisks). The glial cells in the control were intensely labeled with both the β-tubulin antibody (green) and the GFAP (red) antibody. However cells that were treated with AgNPs at 10 μg/ml compromised neuronal soma morphology (indicated by a solid arrow) as well as the neurites (indicated by an open arrow). Notably, the fluorescent intensity of red GFAP and green β-tubulin in glial cells (indicated by asterisks) was remarkably affected by AgNPs (Figure 5C). In summary, our data demonstrate that the major components of neuronal and glial cytoskeletal proteins, β-tubulin and F-actin, were affected by AgNPs.

### AgNPs induce alterations in pre- and postsynaptic proteins in rat cortical neurons

**Figure 6:**
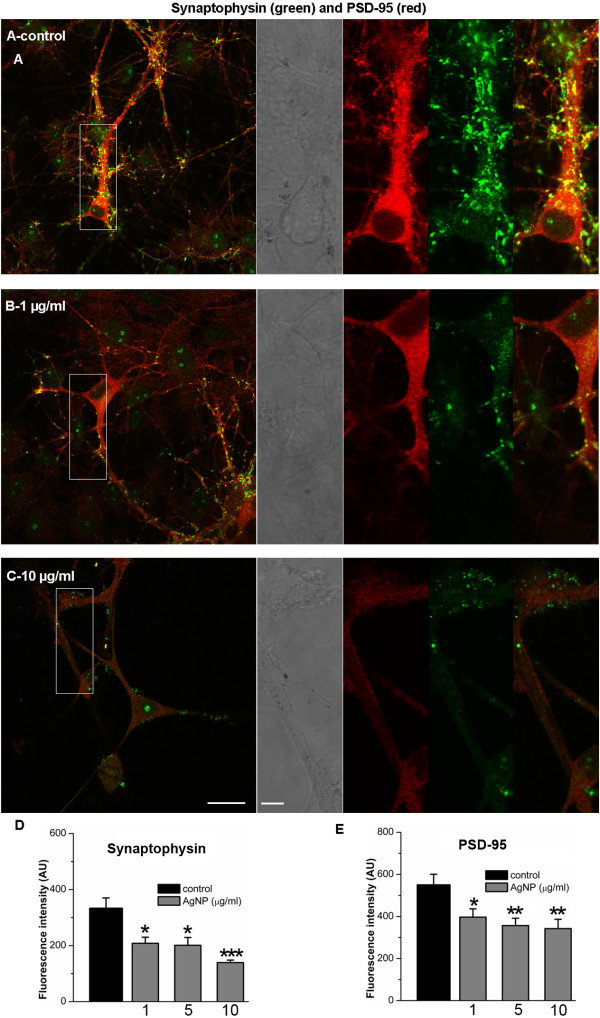
**Immunostaining study of the presynaptic vesicle membrane protein synaptophysin (green), and the postsynaptic marker PSD-95 (red) in control and AgNP-treated neurons.** To examine how AgNPs affect synaptic structural and functional components, cells were first cultured for 10 days and allowed to develop synaptic structures. Cells were then exposed to AgNPs at 1, 5, and 10 μg/ml for another 2 days. Neurons were then incubated with antibodies against synaptophysin and PSD-95. Neurons kept under control culture conditions (**A**, also see inserts) showed extensive neuritic processes in which dense puncta labeling of the synaptic vesicle protein synaptophysin (green) was observed. In addition, both cell bodies and dendritic neurites exhibit high intensity of red fluorescent labeling of PSD-95. Co-localized spots (yellow) represent the location of potential synapses. However, AgNP treatment even at low concentration of 1 μg/ml drastically reduced both the green fluorescent synaptophysin and red fluorescent PSD-95 (**B**). The reduction in fluorescent intensity of synaptic proteins by AgNPs at the 10 μg/ml was accompanied by injured cell morphology and compromised network integrity (**C**). The data shows that the mean fluorescent intensity of both the synaptophysin (**D**) and PSD-95 (**E**) was significant decreased by AgNPs at the concentrations examined. Scale bars, 20 μm (**A**,**B**,**C**) and 5 μm (inserts).

Cytoskeletal components not only serve as fundamental structures for cell integrity and survival, but also play a pivotal role in supporting, organizing and trafficking of synaptic elements, which are crucial for neuronal communication and synaptic plasticity [19-21]. Thus, the disturbance of cellular cytoskeletal components may thus have an effect on synaptic structures and functions. To this end, we investigated whether AgNPs could affect synaptic proteins such as synaptophysin, a presynaptic vesicle protein, and PSD-95, a postsynaptic density protein, that play important roles in synaptic transmission, synapse maturation and synaptic plasticity [22,23]. To do this, we cultured cortical neurons for 10 days and allowed for the establishment of a network and maturation of synaptic components. Cells were then exposed to culture medium either in the absence (control) or presence of AgNPs at different concentrations for 2 days. The effect of AgNPs on the expression level and clustering of synaptic proteins, synaptophysin and PSD-95 were examined on day 12 using immunofluorescence staining and confocal microscopy. Figure 5 shows that control neurons displayed extensive dendritic arborization and mature synaptic contacts, evident by distinct punctate labeling with a presynaptic marker of synaptophysin and a postsynaptic marker of PSD-95 (also see inserts). Cells exposed to AgNPs even at 1 μg/ml revealed a remarkable decline in the density of fluorescence buttons, although they maintained a integrative somatic and neuritic morphology, indicating the degradation of synaptic proteins of both synaptophysin and PSD-95 (Figure 5B, also see insert). AgNPs, at a high concentration of 10 μg/ml, induced not only severe damage to the neuronal network integrity but also robust degradation of synaptic proteins (Figure 5C, also see insert). Because of the severe deficit in neuronal structural integrity at high concentrations of AgNPs, it was difficult to not only distinguish dendrites from axons, but also to quantify the density of buttons in the same length of dendrites. We were however able to measure and compare the mean fluorescent intensity of neuronal synaptophysin and PSD-95 in areas of 1340 μm^2^ under control and AgNP-treated conditions. Our quantified data from four repeated experiments demonstrated that the fluorescent intensity of both presynaptic synaptophysin and postsynaptic PSD-95 was significantly reduced by AgNPs at all concentrations examined (Figure 6D and 6E). Specifically, the level of fluorescent intensity of synaptophysin under control conditions was 333.3 ± 36.7 AU (n = 13) and it was reduced to 208.2 ± 21.5 AU (n = 12, P = 0.04) by 1 μg/ml of AgNPs, to 201.0 ± 28.0 AU (n = 12, P = 0.04) by 5 μg/ml of AgNPs, and to 139.3 ± 8.8 AU (n = 12, P < 0.001) by 10 μg/ml of AgNPs. Similarly, the level of fluorescent intensity of PSD-95 under control conditions was 550.0 ± 50.3 AU (n = 13) and it was reduced to 396.8 ± 39.2 AU (n = 12, P = 0.03) by 1 μg/ml of AgNPs, to 356.4 ± 35.1 AU (n = 12, P = 0.005) by 5 μg/ml of AgNPs, and to 342.2 ± 44.28 AU (n = 12, P = 0.002) by 10 μg/ml of AgNPs.

### Assessment of mitochondrial integrity after AgNP treatment

**Figure 7:**
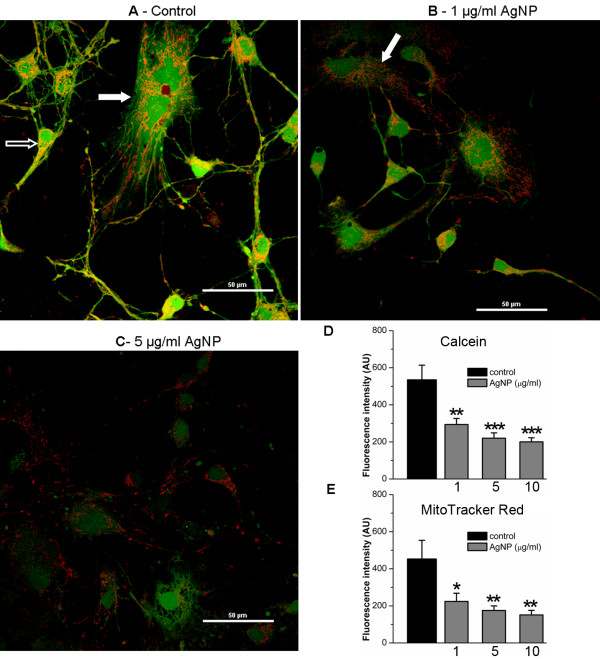
**Cell viability and mitochondria staining of rat cortical cultures under control and AgNP-treated conditions.** Cells were first cultured for 4 days and subsequently exposed to AgNPs at 1, 5, and 10 μg/ml for another 2 days. On day 6, MitoTracker Red, a selective mitochondria marker and calcein AM, an indicator of cell viability, were loaded in control and AgNP-treated cultures. Representative images show that cells maintained in control culture medium (**A**) displayed intense green fluorescence staining of healthy structures of all cell types including neurons (indicated by open arrows) and glial cells (indicated by solid arrows). These cells also contain intense and well-organized labeling of mitochondrial structures by the red fluorescent MitoTracker Red dye. Co-localized spots (yellow) likely represent the location of intact and functional mitochondria. However, AgNP-treated cells (**B** and **C**) drastically lost the green fluorescence intensity and a degree of yellow staining indicating that AgNPs induced decrease of cell viability is accompanied by mitochondrial dysfunction. Statistical analysis shows that the fluorescent intensity of both calcein (**D**) and MitoTracker Red (**E**) was reduced significantly by AgNPs. Statistical significance was determined using ANOVA one-way analysis of variance. Post hoc analysis was conducted using Tukey’s test. * P < 0.05. ** P < 0.01. *** P < 0.001. Error bars indicate SEM.

There is increasing evidence linking mitochondria to cell death, abnormal cytoskeletal and synaptic proteins in a variety of cell preparations [24-26]. To assess whether AgNPs affect the integrity and function of mitochondria, we simultaneously stained cells with MitoTracker Red, a specific marker for mitochondria, and calcein AM, an indicator of cell viability. To do this, cells were cultured in the absence of AgNPs for 4 days, which allowed for development of healthy neurites and networks. Cells were then exposed to AgNPs for another 2 days. On day 6, cells were loaded with MitoTracker Red and calcein AM for 15 mins at 37°C. Fluorescent images were acquired using confocal microscopy (Nikon, USA) under a 60× water objective. In viable cells, calcein AM enters cell cytosol as well as in the mitochondria and emits green fluorescence in both structures. The MitoTracker Red selectively marks the presence of mitochondria and its uptake relies upon potential gradient across mitochondrial membrane. The overlapping yellow color indicates simultaneous and active intake of both dyes and the amount of fluorescent intensity indicate the functional degree of mitochondria in the uptake of these two dyes. As shown in Figure 7A, control neurons (indicated by open arrows) and glial cells (indicated by solid arrows) reveal healthy cell bodies and neuronal processes displayed by the intense green fluorescence of calcein and MitoTracker Red in these structures. The intensive and high incidence of co-labeling (yellow) of mitochondria designates the functional integrity of mitochondria in control cells (Figure 7A). Our data analysis from four experiments revealed that under control conditions, the green fluorescent intensity of calcein in areas of 3800 μm^2^ was 534.6 ± 79.1 AU (n = 8) and the red fluorescent intensity of MitoTracker Red was 452.8 ± 100.8 AU (n = 8) (Figure 7D &7E). The fluorescent intensity of both calcein and MitoTraker Red dyes was however significantly reduced by 1 μg/ml of AgNPs to 293.7 ± 33 AU (n = 9, P = 0.003) and 223.9 ± 44.4 AU (n = 9, P = 0.02), respectively. Specifically, cells exposed to 1 μg/ml of AgNPs for 2 days exhibited compromised cell morphological integrity in combination with reduced intensity of green and red fluorescence (Figure 7B). The absence of green fluorescence in MitoTracker Red-labeled mitochondria (indicated by solid arrows) indicated the lack of active esterase in mitochondria or a leak of green calcein from injured mitochondria (Figure 7B). The minimum co-localization of green and red in higher concentrations of AgNPs (e.g. 5 μg/ml, Figure 7C)-treated cells further indicated the detrimental effect of AgNPs on mitochondrial functionality. As shown in Figure 7D and 7E, the fluorescent intensity of calcein was significantly reduced to 220.1 ± 28.8 AU (n = 11, P < 0.001) by 5 μg/ml of AgNPs and to 200.4 ± 21.9 AU (n = 11) by 10 μg/ml of AgNPs. The fluorescent intensity of MitoTracker Red was reduced to 175.4 ± 24.5 AU (n = 11, P = 0.003) by 5 μg/ml of AgNPs and to 151.1 ± 25 AU (n = 11) by 10 μg/ml of AgNPs.

## Discussion

The present study provides the first morphological and cellular evidence that exposure to AgNPs results in a reduction in synaptic proteins, cytoskeletal integrity, mitochondria functionality and cell viability in a dose-dependent manner. Specifically, AgNPs not only inhibited neurite extension and overlap during the early stage of neuronal development, but also caused degeneration of neuritic processes or aberrant aggregations of cell bodies in well-established neurons and their networks. AgNPs-induced neurotoxicity involved altering cytoskeletal proteins (e.g. β-tubulin and F-actin), dissolution of synaptic proteins (e.g. synaptophysin and PSD-95), and compromising of mitochondria function. Our data show that the AgNP – induced reduction of cellular viability occurs in all types of cells in the primary culture. Recent awareness has raised concerns regarding the impact of environmental factors (e.g. heavy metals, pesticide etc) on human and animal health involving a wide range of diseases including cancer, liver, lung, and kidney diseases as well as brain disorders. Our study together with several published accounts thus cautions against the chronic and extensive use of AgNPs in products that may come in direct contacts with all living organisms [7,13-15].

With the noteworthy maturation of nanotechnology during the last decade, nanoparticle products will continue to be used increasingly in our everyday commercial products, industrial processes and medical applications. Such extensive and unregulated exposure to ultrafine-size substances released either into the atmosphere and/or water system, food and therapeutic products holds potential hazardous risks not only to humans but also to all organisms [27-30]. Recent studies have revealed that AgNPs, one of the most commonly used metal nanoparticles, cause severe developmental deficits not only in aquatic animals but also aquatic plants [31,32]. Owing to their antibacterial properties, AgNPs have been predominately used for the development of medicines, drug delivery systems and medical device coatings [4,7]. AgNPs can be translocated to the blood stream and distributed throughout vital organs such as the liver, kidney, lung and brain [4,5,11]. AgNPs can cross the BBB and cause BBB inflammation and increase in permeability indicating the potential risk for toxicity to the brain [5,10,12]. While the literature is silent on the precise concentrations of AgNPs found in the brain as it crosses the BBB, several studies have reported that the rate of nanoparticle translocation into the brain can be significantly increased under certain pathological conditions, such as infection, meningitis, systemic inflammation etc [8,13]. Our choice of AgNP concentration and size was based on previously established works. AgNPs (20 nm) were used due to their known high cytotoxic properties with respect to permeating and damaging cerebral microvascular structures, as compared with larger particles (40 nm and 80 nm) [12]. Similarly, smaller nanoparticles (20 nm) have also been shown to induce higher levels of cellular oxidative damage [16]. Previous experiments conducted with 20-40 nm AgNPs used concentrations ranging from 1 to 100 μg/ml to examine the potential hazardous effects of AgNPs with primary neuronal cells [16-18,33]. In these studies, AgNPs have been found to inhibit neuronal sodium and potassium currents at 10 μg/ml, disturb neuronal calcium homeostasis at 5 μg/ml, and reduce dopamine concentration at 50 μg/ml. Based on these studies, we pursued to identify the effects of chronic exposure to AgNPs (20 nm) at the low to medium doses of 1, 5, 10 and 50 μg/ml on the primary rat cortical cell viability, cytoskeletal frameworks, key synaptic proteins, and mitochondrial function.

Because all brain functions rely critically upon the normal development of neuronal structures and network circuitry, any perturbation of these processes will render the nervous system dysfunctional. In the course of neuronal development, cell viability and neurite outgrowth are two fundamental and essential factors enabling neurons to reach their potentials targets and establish functional communications. These steps rely upon the integrity of cytoskeletal structures. Specifically, cytoskeletal components play imperative roles in neuronal architecture formation and maintenance such as neurite outgrowth, axon guidance, information transmission, and functional synaptic circuitry establishment [34-36]. The reduced number of stained branches and neurite processes by β-tubulin and F-actin antibodies in cells cultured in the presence of AgNPs indicate that AgNPs may inhibit neurite initiation and sprouting by disturbing the assembly/disassembly of these cytoskeletal proteins. The intact cytoskeleton also plays an indispensible role in the localization and trafficking of synaptic machinery (neurotransmitters/receptor proteins) and intracellular organelles including the mitochondria. The degeneration of microtubule and neurofilament components by AgNPs in a well-established neuronal network may directly or indirectly contribute to AgNP-induced loss of synaptic proteins (eg. PSD-95 and synaptophysin, Figure 6) and injury of mitochondria (Figure 7). One would need to be aware that AgNPs might first cause impairment of synaptic proteins and/or mitochondria which may in turn, deprive the fundamental cytoskeletal structures of their vitality source and hence the ensuring collapse. Future studies using time-lapse imaging in combination with fluorescent tagging techniques will be required to investigate these possibilities further. Nevertheless, our data does indicate that by curtailing the growth patterns of neurons, AgNP may not only prevent normal brain development but also preclude neuronal plasticity, learning and memory that relies upon new growth of process and synapses.

We found that exposure to AgNPs exerted detrimental effects on all types of cells including glial cells present in the primary, rat cortical cultures. It is well appreciated that glial cells not only play crucial supportive roles in the development and maintenance of neuronal structures, but that they also actively communicate with neurons to enable synapse formation, synaptic transmission, plasticity and synaptic homeostasis [37,38]. The pronounced loss of glial cells or their ability to form layers in AgNP-treated cultures (Figure 3) indicates that the glial cell morphology can be severely compromised by AgNPs. This finding is further supported by our immunostaining of glial cell cytoskeletal β-tubulin and GFAP (Figure 5C) in which glial microtubules and intermediate filament proteins were compromised in AgNP-treated culture, while intense green labeling of glial β-tubulin and GFAP was evident in control cultures. This data suggests that components of the glial cytoskeleton are the major target for AgNP-induced toxicity in glial cells. Our findings are consistent with a previous study demonstrating that the glial cell astrocytes were more sensitive to AgNP insult than neurons [16]. Glial cells also play guiding and adhesion roles during neuronal network formation [39-41], hence any absence of preferred neuronal adhesion to glia may contribute to AgNP-induced aggregation of cell bodies and neurofibrillary processes that were observed in our study (Figure 3G and 3H). In addition, a lack of proper innervations and communications between neurons and neuroglia may also markedly deprive them of their glial trophic support. Moreover, in the absence of glial neuro-protection, the neurotoxic effects of AgNPs may have also been exacerbated. These in turn may also impact synaptic transmission, deteriorate synaptic components, and eventually lead to cell death. Synaptic damage has been implicated in a variety of brain disorders, including traumatic nerve injury, stroke, and many neurodegenerative disorders, such as Alzheimer’s, Parkinson’s and Huntington’s diseases [42-44]. Alterations to synaptic structures rank among the earliest notable features in the commencement of the cognitive decline characterized and represented in the co-morbidity of Alzheimer’s diseases [45]. In fact, studies have shown that normal animals treated with AgNPs exhibited reduced cognitive/motor functions and altered cellular structures in the brain [13]. This study together with our data showing the AgNP toxicity to nervous tissues at the cellular, molecular and system levels during both developing and mature stages advise against the potential chronic exposure to AgNPs not only in young people whose brains undergo rapid development, but also in adults whose cognitive functions require continued growing of new neuronal networks.

## Conclusions

Taken together, this study demonstrates the potentially devastating effects of AgNPs on cell survival, synaptic protein localization, cytoskeletal proteins, and mitochondrial integrity. Based on the fact that AgNPs have emerged as an important class of nano materials for a wide range of industrial and medical applications, our study highlights not only an urgent need to assess their potential health risks at the whole organism level but also to develop strict policies for their usage.

## Methods

### Rat cortical cultures

All animal procedures were approved by the University of Calgary Animal Care Committee. Conditions met with the standards established by the Canadian Council on Animal Care. The primary culture of rat cortical cells was made using Sprague–Dawley rat pups at postnatal day zero. Dissociated cortical neurons were plated onto cover slips coated with poly-D-lysine (30 μg/ml, Sigma P6407) and Laminin (2 μg/ml, Sigma L2020). Cortical neurons were cultured in neurobasal medium (Invitrogen, no. 21103-049) supplemented with 2% B27 (Invitrogen, no. 17504-044), L-Glutamine (200 mM) (Invitrogen, no. 25030-081), 4% FBS (Invitrogen, no. 12483-020), and penicillin-streptomycin (Invitrogen, no. 15140-122). Approximately 80% of the culture media was replaced every 3-4 days. Cultures were maintained at 37°C in an incubator circulated with air and 5% carbon dioxide.

To study the effect of AgNPs on neuronal process initiation, neurite outgrowth and overlap, freshly dissociated cortical neurons were cultured either in the absence (control) or presence of different concentrations of AgNPs (1, 5, 10 and 50 μg/ml) for 3 days. Neurite outgrowth and cell viability were evaluated on day 3. To examine the effect of AgNPs on newly established neuronal processes and overlaps, cells were first cultured for 4 days to allow for the establishment of neurite outgrowth and network. Neurons were then exposed to AgNPs at different concentrations for 2 days, and the effects of AgNPs were examined. To study the effect of AgNPs on well-established mature network, 10 day-old cells cultured on cover slips were exposed to different concentrations of AgNPs for two days and the effects were evaluated on day 12.

### Live/dead cell viability assay for cortical cells

To determine and quantify the impact of AgNPs on cell viability, cortical cultures that were maintained under control or drug-treated conditions were subsequently loaded with the LIVE⁄DEAD^®^ Viability⁄Cytotoxicity Kit (Molecular probes, L-3224) for 15 mins at room temperature (21-22°C). This two-color assay was developed based on the fact that intracellular esterase activity and an intact plasma membrane are unique characteristics of live cells. The LIVE⁄DEAD^®^ Viability⁄Cytotoxicity Kit discriminates live from dead cells by simultaneously staining with green-fluorescent calcein-AM to indicate intracellular esterase activity and red-fluorescent ethidium homodimer-1 to indicate loss of plasma membrane integrity. Preparations were visualized using confocal microscopy (LSM 510 Meta, Zeiss, Germany) under a 20× objective at 488 nm excitation (green) and 548 nm (red) wavelength and images were collected using a band pass filter (560-615 nm). The number of cells labeled with both colors was subsequently counted using imageJ software.

### Immunochemistry and confocal microscopy

To stain the cytoskeletal proteins of β-tubulin and F-actin, cultured cells were fixed for 1 h with pre-warmed 4% paraformaldehyde and subsequently washed four times with 1× PBS and permeabilized for 1 h with incubation media (IM) (0.5% Triton in 1× PBS with 10% goat serum). Preparations were then incubated overnight at 4°C with a monoclonal anti-β-tubulin antibody produced in mouse (1:500) (Sigma, T0198). The next day, cells were rinsed twice with 1× PBS and incubated with Alexa Fluor^®^ 488 goat anti-mouse IgG secondary antibody (1:100) (Invitrogen, A-11001) for 1 h at room temperature (21-22°C) under dark conditions. Cultures were subsequently rinsed two times with 1× PBS and incubated for 30 minutes with rhodamine phalloidin (1:20) (Invitrogen, R415) at room temperature. Following two washes with 1 × PBS and one quick rinse with double distilled H_2_O, cover slips were mounted using MOWIOL mounting media with 4′6-diamidino-2-phenylindole dihydrochloride (Sigma-Aldrich). Samples were viewed using confocal microscopy (LSM 510 Meta, Zeiss, Germany) under a 20× or 63× oil objective at 488 nm (green, β-tubulin) and 548 nm (red, F-actin) excitation wavelengths. Images were collected using a band pass filter (560-615 nm). To stain glial cytoskeletal proteins, the mouse monoclonal anti-β-tubulin antibody (1:200) (Sigma, T0198) and rabbit monoclonal anti-GFAP antibody (1:200) (Biomedical Technologies Inc., BT-575) were stained following procedures as described above. The secondary antibodies were Alexa Fluor^®^ 488 goat anti-mouse IgG antibody (1:100) (Invitrogen, A-11001) and Alexa Fluor^®^ 546 goat anti-rabbit IgG antibody (1:100) (Invitrogen, A-11010). Image acquisition parameters (e.g. laser intensity, gain settings, pinhole sizes, exposure time etc) for control and drug-treated neurons were kept the same. Negative control experiments were performed at the same time to test the specificity of ß-tubulin, F-actin, and GFAP antibodies. No immunofluoresecence was detected when primary antibodies were excluded from the staining procedures (see Additional file 1A and 1B). To verify whether neurons exhibited autofluorescence, unstained cells were excited with all lasers (633, 488, 546 nm) of the confocal microscope and no autofluorescence was observed (data not shown).

To stain the synaptophysin and PSD-95, cultured cells were fixed with pre-warmed 4% paraformaldehyde and 15% picric acid at room temperature (21-22°C) for 20 mins. Cells were subsequently washed four times with 1× PBS and permeabilized for 1 h with blocking incubation media (0.1% Triton in 1× PBS with 5% goat/donkey serum and 2% BSA). Preparations were then incubated overnight at 4°C with the monoclonal anti-PSD-95 antibody produced in mouse (1:2000) (NeuroMab, 75-028) and an anti-synaptophysin monoclonal antibody produced in rabbit (1:500) (Abcam, Ab52636). The next day cells were rinsed twice with 1× PBS. Cells were then incubated with Alexa Fluor^®^ 488 goat anti-rabbit IgG secondary antibody (1:100) (Invitrogen, A-11001) and Alexa Fluor^®^ 546 goat anti-mouse IgG secondary antibody (1:100) (Invitrogen, A11030) for 1 h at room temperature (21-22°C) under dark conditions. Following two washes with 1× PBS and one quick rinse with double distilled H_2_O, cover slips were mounted using MOWIOL mounting media with 4′6-diamidino-2-phenylindole dihydrochloride (Sigma-Aldrich). Samples were viewed using confocal microscopy (LSM 510 Meta, Zeiss, Germany) under a 63× oil objective at 488 nm excitation (green, synaptophysin) and 548 nm (red, PSD95) wavelength. Images were collected using a band pass filter (560-615 nm). To assess the level of synaptophysin and PSD-95 staining, image acquisition parameters (laser intensity, gain settings, pinhole sizes, exposure times etc) for control and drug-treated neurons were kept the same. As a specificity control for the immunostaining, no immunofluorescence was observed when primary antibodies of synaptophysin and PSD-95 were excluded from the above staining procedures (see Additional file 1C and 1D).

To assess the impact of AgNPs on the integrity and function of mitochondria, cortical cultures were first maintained in control medium for 4 days and cells were then exposed to AgNPs for another 2 days. On day 6, control or drug-containing medium was subsequently washed off twice with warm Hanks’ Balanced Salt Solution (HBSS) containing sodium bicarbonate, calcium, and magnesium that also included HEPES (10 mM), L-glutamine (2 mM) and succinate (100 μM) to support healthy mitochondrial function in live cells. Cells were then incubated in a dye-loading solution containing calcein AM (1 μM) and MitoTracker Red CMXRos (200 nM) for 15 mins at 37°C. Fluorescence images of mitochondrial integrity (red) and cell viability (green) were collected using a Nikon Eclipse C1si Spectral Confocal microscope with motorized stage (Nikon Instruments Inc., Melville, NY, United States) at the excitation wavelength of 561 nm (red, with a 590/50 emission filter) and 488 nm (green, with a 515/30 emission filter) through a 60× water objective. Again, image acquisition parameters for control and drug-treated neurons were kept the same.

### Chemicals

Silver nanoparticles (AgNPs) were purchased from nanoComposix (Sandiego, CA, USA). All chemicals were purchased from Sigma-Aldrich (Oakville, Ontario, Canada).

### Statistical analysis

Data was analyzed statistically using one-way analysis of variance (ANOVA) as appropriate. Post hoc analysis was conducted using Tukey’s test. Values were considered statistically significant at the level of P < 0.05. The data is presented as mean ± S. E.M. Each experiment was replicated a minimum of four times; the actual number of replicates for each experiment is listed in the corresponding figure legend or in the text.

## Competing interests

The authors declare that they have no competing interests.

## Authors’ contributions

FX and CP initiated the project and conducted data analysis. FX, CP, SF, and MQ performed experiments. Specifically, FX, SF, and CP performed the cell viability, cytoskeleton, and synaptic protein studies. FX, MQ, and SF conducted the mitochondria study. FX and NIS drafted and edited the manuscripts. All authors read and approved the final manuscript.

## Supplementary Material

Additional file 1 **Specificity of antibody staining.** To test for the specificity of antibodies (β-tubulin, GFAP, synaptophysin and PSD-95) staining, control experiments in which the primary antibodies were excluded were performed at the same time following procedures as described in the method section. When primary antibodies were excluded from the staining procedure, no immunofluorescence was observed (B- without β-tubulin/GFAP; D-without synaptophysin/PSD-95). In contrast, intense immunofluorescence was detected when primary antibodies were included (A-with β-tubulin/GFAP; C- with synaptophysin/PSD-95). Note that because all the antibodies used were monoclonal (as described in the Methods), we therefore did not perform antigen pre-absorption test for polycolonal antibodies. Click here for file
