# Supplementary material for: Silver nanoparticles (AgNPs) cause degeneration of cytoskeleton and disrupt synaptic machinery of cultured cortical neurons
Source: Mol Brain. 2013 Jun 19;6:29. doi: 10.1186/1756-6606-6-29 (PMC3695839; doi:10.1186/1756-6606-6-29)
Supplement: Additional file 1 — Specificity of antibody staining. To test for the specificity of antibodies (β-tubulin, GFAP, synaptophysin and PSD-95) staining, control experiments in which the primary antibodies were excluded were performed at the same time following procedures as described in the method section. When primary antibodies were excluded from the staining procedure, no immunofluorescence was observed (B- without β-tubulin/GFAP; D-without synaptophysin/PSD-95). In contrast, intense immunofluorescence was detected when primary antibodies were included (A-with β-tubulin/GFAP; C- with synaptophysin/PSD-95). Note that because all the antibodies used were monoclonal (as described in the Methods), we therefore did not perform antigen pre-absorption test for polycolonal antibodies. [file 1756-6606-6-29-S1.pdf]

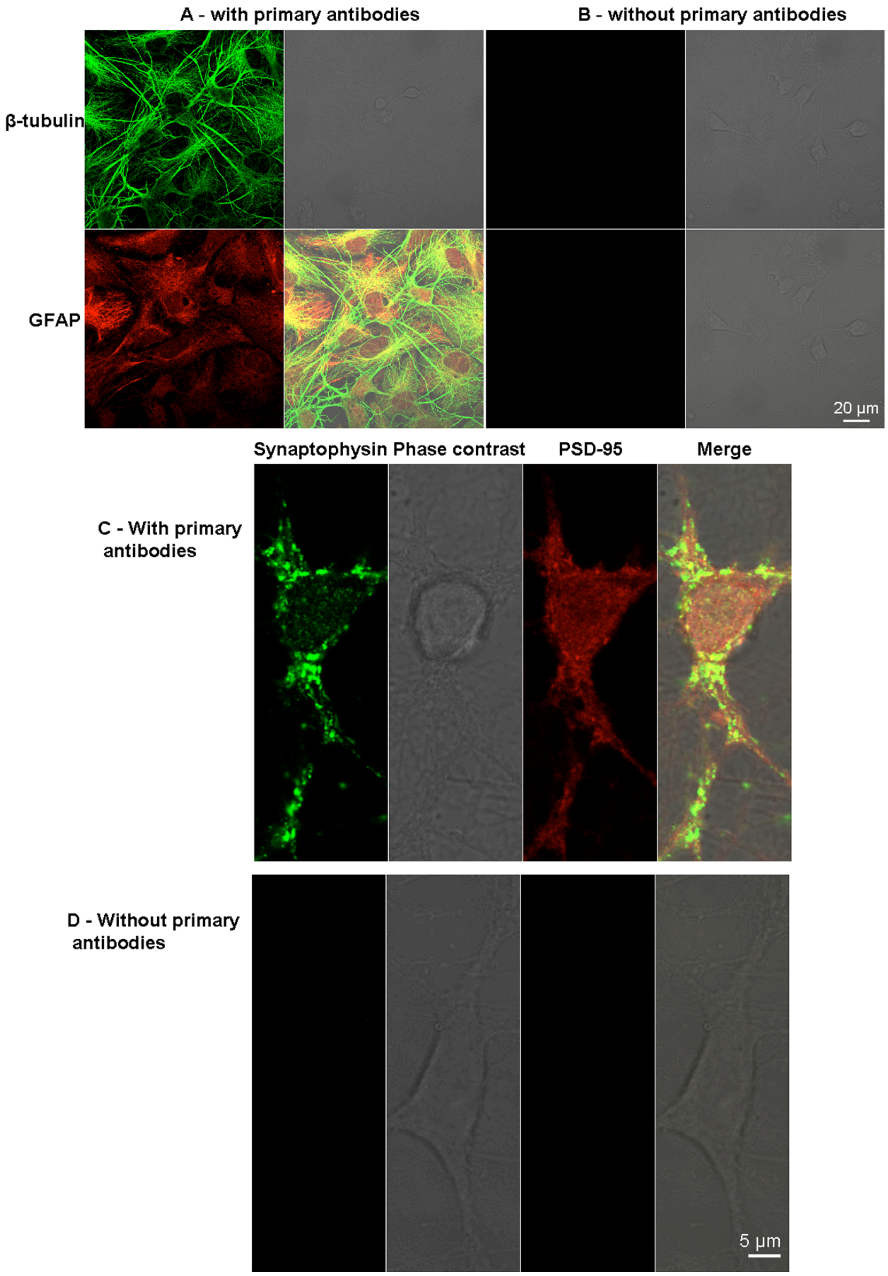


**Additional file 1: Specificity of antibody staining.** To test for the specificity of antibodies (β-tubulin, GFAP, synaptophysin and PSD-95) staining, control experiments in which the primary antibodies were excluded were performed at the same time following procedures as described in the method section. When primary antibodies were excluded from the staining procedure, no immunofluorescence was observed (***B***- without β-tubulin/GFAP; ***D***-without synaptophysin/PSD-95). In contrast, intense immunofluorescence was detected when primary antibodies were included (***A***-with β-tubulin/GFAP; ***C***- with synaptophysin/PSD-95). Note that because all the antibodies used were monoclonal (as described in the methods), we therefore did not perform antigen pre-absorption test for polycolonal antibodies.

***Additional file 1 Xu et al.***
